# Cefdinir Solid Dispersion Composed of Hydrophilic Polymers with Enhanced Solubility, Dissolution, and Bioavailability in Rats

**DOI:** 10.3390/molecules22020280

**Published:** 2017-02-13

**Authors:** Hyun-Jong Cho, Jun-Pil Jee, Ji-Ye Kang, Dong-Yeop Shin, Han-Gon Choi, Han-Joo Maeng, Kwan Hyung Cho

**Affiliations:** 1 College of Pharmacy, Kangwon National University, 1 Kangwondaehak-gil, Chuncheon 24341, Korea; hjcho@kangwon.ac.kr; 2 College of Pharmacy, Chosun University, 309 Pilmun-daero, Gwangju 61452, Korea; jee@chosun.ac.kr; 3 College of Pharmacy, Inje University, 197 Inje-ro, Gimhae 50834, Korea; tmxhq0818@naver.com; 4 School of Pharmacy, Sungkyunkwan University, 300 Cheoncheon-dong, Jangan-gu, Suwon 16419, Korea; dyshin73@gmail.com; 5 College of Pharmacy & Institute of Pharmaceutical Science and Technology, Hanyang University, 55 Hanyangdaehak-ro, Ansan 15588, Korea; hangon@hanyang.ac.kr; 6 College of Pharmacy, Gachon University, 191 Hambakmoei-ro, Yeonsu-gu, Incheon 21936, Korea

**Keywords:** cefdinir anhydrous, solid dispersion, hydrophilic polymers, dissolution, bioavailability, rats

## Abstract

The aim of this work was to develop cefdinir solid dispersions (CSDs) prepared using hydrophilic polymers with enhanced dissolution/solubility and in vivo oral bioavailability. CSDs were prepared with hydrophilic polymers such as hydroxypropyl-methylcellulose (HPMC; CSD1), carboxymethylcellulose-Na (CMC-Na; CSD2), polyvinyl pyrrolidone K30 (PVP K30; CSD3) at the weight ratio of 1:1 (drug:polymer) using a spray-drying method. The prepared CSDs were characterized by aqueous solubility, differential scanning calorimetry (DSC), powder X-ray diffraction (p-XRD), scanning electron microscopy (SEM), aqueous viscosity, and dissolution test in various media. The oral bioavailability of CSDs was also evaluated in rats and compared with cefdinir powder suspension. The cefdinir in CSDs was amorphous form, as confirmed in the DSC and p-XRD measurements. The developed CSDs commonly resulted in about 9.0-fold higher solubility of cefdinir and a significantly improved dissolution profile in water and at pH 1.2, compared with cefdinir crystalline powder. Importantly, the in vivo oral absorption (represented as AUC_inf_) was markedly increased by 4.30-, 6.77- and 3.01-fold for CSD1, CSD2, and CSD3, respectively, compared with cefdinir suspension in rats. The CSD2 prepared with CMC-Na would provide a promising vehicle to enhance dissolution and bioavailability of cefdinir in vivo.

## 1. Introduction

**Figure 1:**
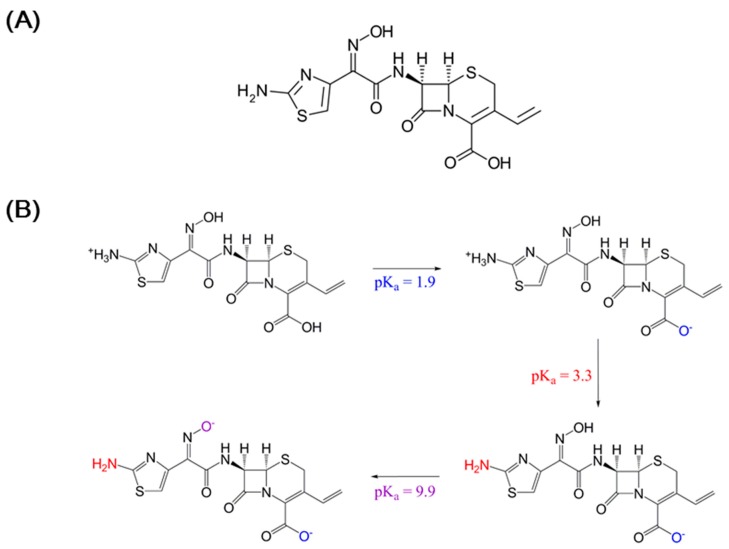
Chemical structure of cefdinir (**A**) and its pH-dependent structural change (**B**).

Cefdinir (8-[2-(2-amino-1,3-thiazol-4-yl)-1-hydroxy-2-nitroso-ethenyl]amino)amino-4-ene-5-carboxylic acid, Figure 1A) is a frequently used third generation oral cephalosporin antibiotic with broad spectrum effectiveness against Gram positive and Gram negative bacteria causing acute chronic bronchitis, rhinosinusitis, and pharyngitis [1]. With respect to the physicochemical properties, the solubility of cefdinir is poor in water (i.e., 0.46 mg/mL) and highly dependent on pH, and likely due to the poor aqueous solubility, it has been reported to have low (approximately 20%) oral bioavailability [2,3], and thus it is classified as a Biopharmaceutics Classification System (BCS) class IV drug with low solubility and low permeability [4]. Therefore, a strategy to enhance solubility/dissolution and ultimately the oral bioavailability of cefdinir would be important in order to improve the therapeutic effect in the body after oral administration. Several recent studies to improve the solubility of cefdinir have been reported in the literature [4,5]. The preparation of cefdinir nanosuspension using a media milling technique improved its oral bioavailability in rats by about 3-fold, compared with a marketed cefdinir suspension (i.e., Omnicef), likely due to increased solubility, dissolution rate and permeation [4]. Consistently, cefdinir-loaded floating formulation with alginate beads demonstrated an enhanced relative bioavailability (by 3.37-fold) compared with cefdinir suspension, supporting with prediction data for specific regional absorption of cefdinir from duodenum and jejunum segment in rabbits [5].

Amorphous solid dispersion is generally a significant formulation strategy to improve the apparent solubility, dissolution rate, and bioavailability of poorly water-soluble drug substance [6]. Drugs in solid dispersions are typically in an amorphous form that is thermodynamically unstable, and has small particle size, eventually providing enhanced aqueous solubility and bioavailability compared with the crystalline form [7]. Amorphous solid dispersions can be achieved by dispersing the poorly water-soluble drug in a biologically inert and pharmaceutically acceptable hydrophilic polymer such as polyvinylpyrrolidone (PVP), hydroxypropylmethylcellulose (HPMC), polyethylene glycol (PEG), polyethylene oxide (PEO), hydroxypropylcellulose (HPC) or carboxymethylcellulose (CMC) [8]. However, the various hydrophilic polymers used as drug carriers in solid dispersions produce different dissolution and bioavailability levels of drug substances since they present different viscosity and mucoadhesiveness values [9,10,11].

The spray-drying method can be applied to obtain amorphous solid dispersions in combination with a hydrophilic polymer [12]. This technology confers the resultant particles with the desirable spherical and free flowing characteristics in a simple drying step [8]. Also, the spray-drying technique has the following advantages: fast and continuous process, applicability to heat-sensitive substances, and easy scale-up.

To the best of our knowledge, no study comparing amorphous solid dispersions composed of cefdinir and various hydrophilic polymers and made via a spray-drying method for the enhancement of bioavailability has been reported yet. The use of different matrix carriers may affect the solubility, particle size, dissolution rate, in particular, and bioavailability of solid dispersion particles. Therefore, in the present study, we developed amorphous cefdinir solid dispersions (CSDs) containing a hydrophilic polymer such as HPMC, CMC, and PVP via a spray drying method. The prepared CSDs were characterized and compared in terms of aqueous solubility, thermodynamic properties, morphological shape, dissolution rate, aqueous viscosity, and in vivo bioavailability.

## 2. Results

### 2.1. Solubility of Cefdinir and CSDs

**Figure 2:**
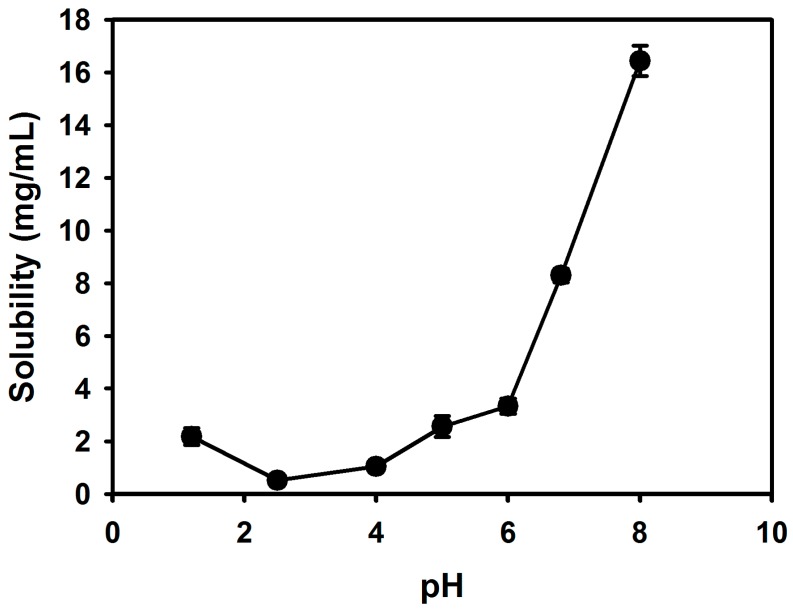
The pH-dependent solubility of cefdinir.

**Figure 3:**
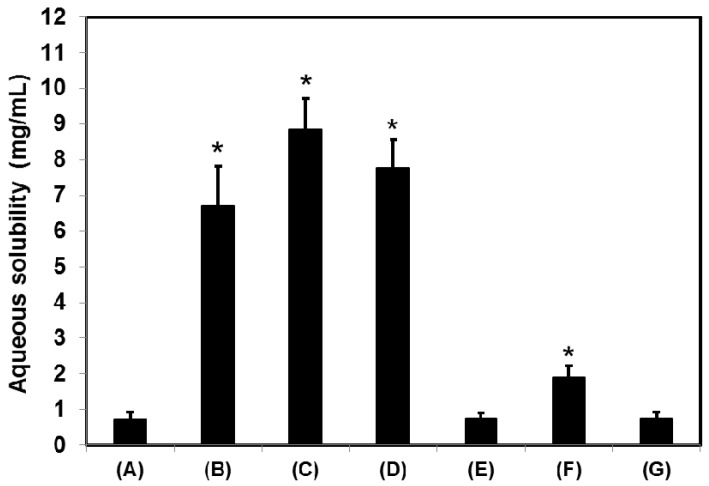
The water solubility of cefdinir in powder (**A**), CSD1 (**B**), CSD2 (**C**), and CSD3 (**D**), physical mixture of cefdinir and HPMC (**E**), physical mixture of cefdinir and CMC-Na (**F**), and physical mixture of cefdinir and PVP K30 (**G**). Data are presented as means ± standard deviation (*n* = 3). * *p* < 0.05, compared with cefdinir in powder group.

Because cefdinir has different forms, depending on the pH (Figure 1B) we studied the solubility of cefdinir anhydrous in aqueous media with different pH values, with the results shown in Figure 2. The resultant solubility was plotted according to the equilibrium solution pH after arriving at saturation. The minimum solubility was shown at pH 2.5 (0.52 ± 0.06 mg/mL) and the solubility was sharply increased over pH 4.0 (16.43 ± 0.58 mg/mL at pH 8.0). The water solubility of cefdinir in physical mixtures with CSDs was also measured (Figure 3). Table 1 summarizes the compositions of the solid dispersion formulations. As a result, the water solubility of cefdinir was significantly enhanced in the CSD formulations (CSD1, 6.71 ± 1.12 mg/mL; CSD2, 8.85 ± 0.87 mg/mL; CSD3, 7.78 ± 0.78 mg/mL), compared with that of physical mixtures or cefdinir itself (0.71 ± 0.21 mg/mL) (*p* < 0.05). Only the physical mixture of cefdinir and CMC-Na displayed a higher solubility compared with that of cefdinir itself or the other physical mixtures.

### 2.2. Solid State Characterization

**Figure 4:**
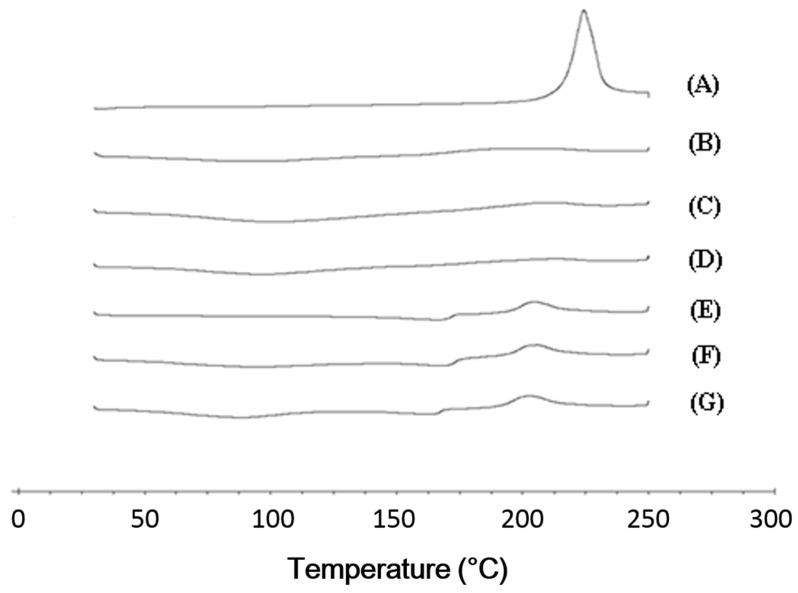
The DSC curves of cefdinir (**A**), CSD1 (**B**), CSD2 (**C**), CSD3 (**D**), physical mixture of cefdinir and HPMC (**E**), physical mixture of cefdinir and CMC-Na (**F**), and physical mixture of cefdinir and PVP K30 (**G**).

**Figure 5:**
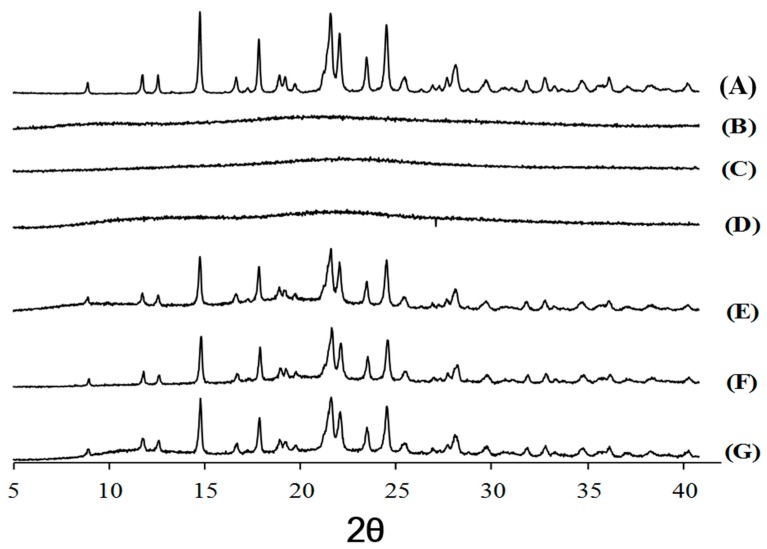
The p-XRD patterns of cefdinir (**A**), CSD1 (**B**), CSD2 (**C**), CSD3 (**D**), physical mixture of cefdinir and HPMC (**E**), physical mixture of cefdinir and CMC-Na (**F**), and physical mixture of cefdinir and PVP K30 (**G**).

**Figure 6:**
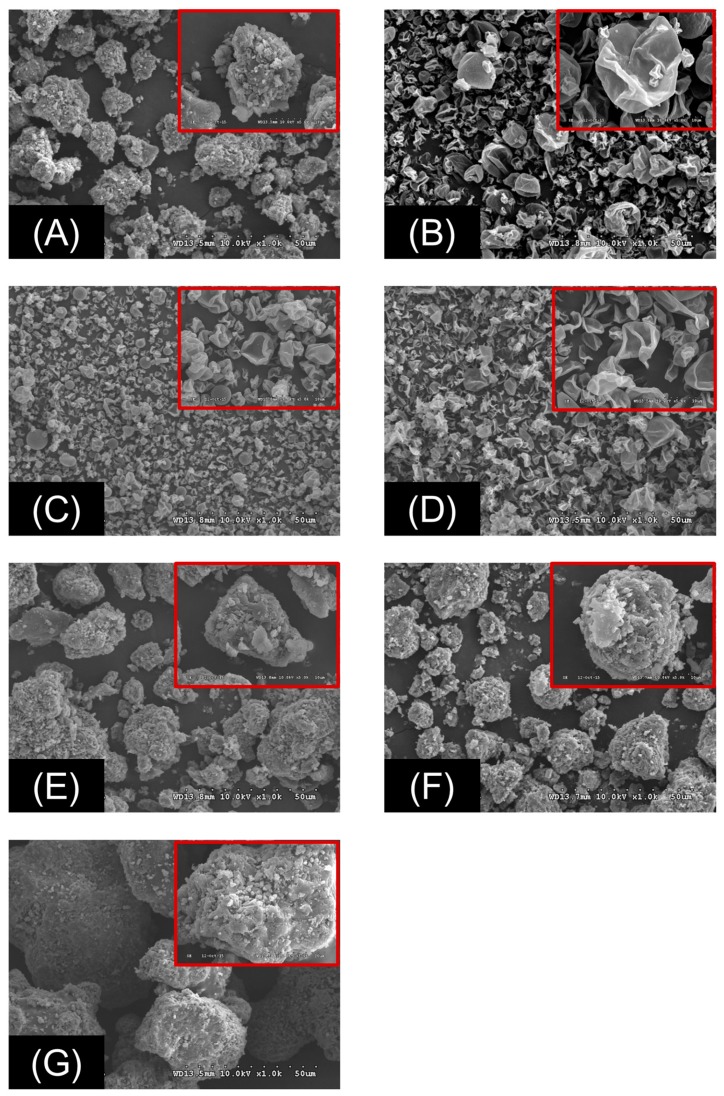
The SEM images of cefdinir (**A**), CSD1 (**B**), CSD2 (**C**), CSD3 (**D**), physical mixture of cefdinir and HPMC (**E**), physical mixture of cefdinir and CMC-Na (**F**), and physical mixture of cefdinir and PVP K30 (**G**). Inset indicates the magnified image.

In the DSC analysis, the cefdinir showed onset and peak point at 216.5 °C and 225.2 °C in the melting curve, respectively (Figure 4A). On the other hand, the peaks indicating melting of cefdinir were absent in the three CSDs (Figure 4B–D), In case of physical mixtures of the drug and a hydrophilic polymer, the intrinsic peak indicating melting of cefdinir, was still present in the DSC curves (Figure 4E–G). The transformation of cefdinir from a crystalline to an amorphous form, was additionally confirmed by p-XRD analysis (Figure 5). Sharp peaks were visible in the profile of cefdinir crystalline form (Figure 5A), but they were absent in the CSD results (Figure 5B–D). Notably, the physical mixtures maintained the diffraction pattern of cefdinir anhydrous (Figure 5E–G). The scanning electron microscopy (SEM) images of cefdinir, CSDs, and physical mixtures of drug and polymer were also obtained, as shown in Figure 6. The agglomeration of small particles with rough surface in the cefdinir group (Figure 6A) was transformed into squeezed particles with smooth surface in CSD groups (Figure 6B–D). The physical mixtures displayed various shaped particles with rough surfaces (Figure 6E–G).

### 2.3. Dissolution and Viscosity Study

**Figure 7:**
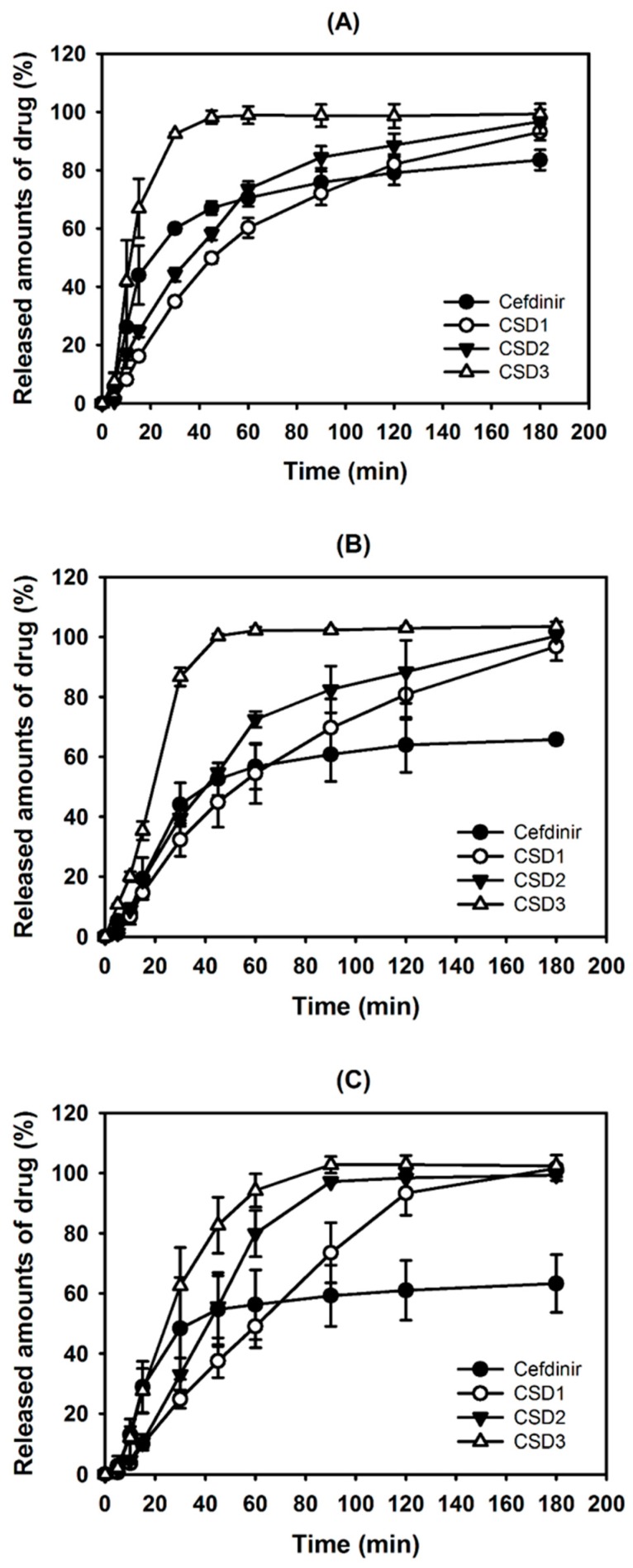
The dissolution curve of cefdinir, CSD1, CSD2, and CSD3 at pH 1.2 (**A**), pH 4.0 (**B**), and water (**C**). Data are presented as means ± standard deviation (*n* = 3).

**Figure 8:**
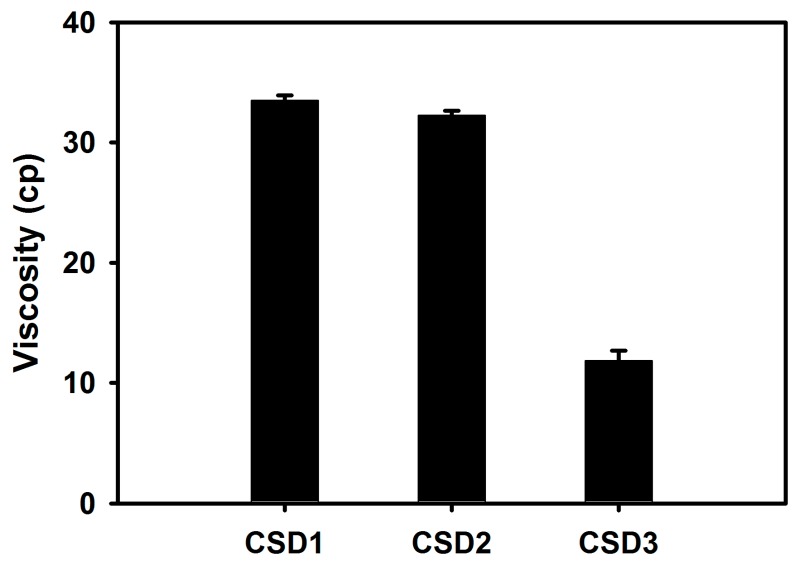
The viscosity of the dispersion of CSD (2%, *w*/*v*) in water. Data are presented as means ± standard deviation (*n* = 3).

The dissolution profiles of cefdinir from CSDs (contained in capsules) were determined and compared with that of cefdinir at pH 1.2 solution, 4.0 buffer, and water (Figure 7). The completion (>95%) of dissolution in CSD1, CSD2, and CSD3 groups was achieved within 180 min. However, the cefdinir group had an incomplete dissolution, as it exhibited an equilibrium phase after 60 min. CSD3 (based on PVP K30) had a higher dissolution rate rather than CSD1 (based on HPMC) and CSD2 (based on CMC-Na). Both CSD1 and CSD2 had similar dissolution curves with steady increments over time, while a dramatic increase was observed in the CSD3 group at both pH 1.2 and 4.0. The viscosities of the CSDs were measured to explain the differences in dissolution profiles (Figure 8). CSD1 (33.51 ± 0.42 cp) and CSD2 (32.26 ± 0.38 cp) had higher viscosities than CSD3 (11.85 ± 0.84 cp) in 2% (*w*/*v*) aqueous solution.

### 2.4. Oral Pharmacokinetic Study

**Figure 9:**
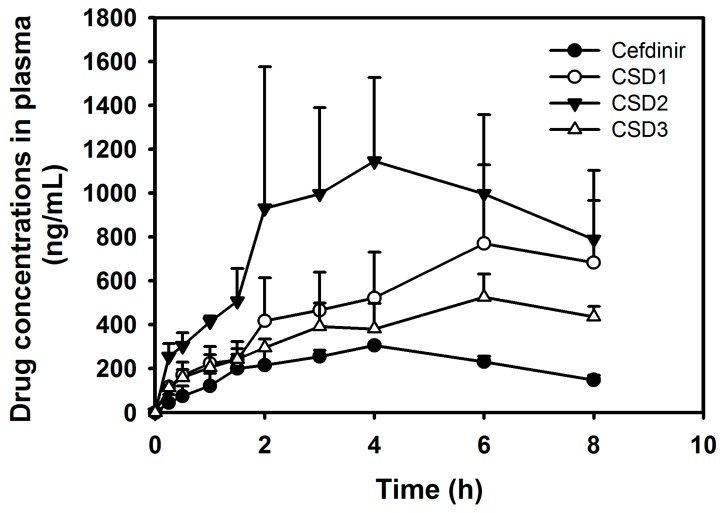
Plasma concentration–time profiles of cefdinir after oral administration of cefdinir and CSDs formulations (CSD1, CSD2, and CSD3) at a dose of 2 mg/kg in rats. Each point represents the mean ± standard error (*n* = 3–4).

To investigate whether the developed CSDs formulations could enhance the oral absorption of cefdinir in vivo, oral pharmacokinetic studies were performed as shown in Figure 9 and Table 2, where the calculated pharmacokinetic parameters for the CSD formulations are summarized, and compared with those of cefdinir group. As a result, the three developed CSDs formulations showed higher plasma concentration-time profiles, compared with the cefdinir group, indicating an enhanced oral absorption of the drug (Figure 9). Interestingly, the C_max_ values of CSD1, CSD2, and CSD3 were significantly increased by 2.52, 4.17, and 1.72-fold, respectively, compared with that of the cefdinir group, while the T_max_ values remained unchanged (Table 2). In addition, the AUC_inf_ values of CSD1, CSD2, and CSD3 were also 4.30, 6.77, and 3.01-fold higher than that of cefdinir group. Among the three CSD formulations, the CSD2 group showed the most enhanced oral absorption in vivo.

## 3. Discussion

Cefdinir is poorly soluble in weak acidic media (pH 2–4), but it is highly soluble over pH 4 (Figure 2). Thus, it showed highly pH-dependent solubility in the tested pH media, which would be a main cause of low oral absorption [2,13]. Cefdinir has three ionizable groups with following p*K*_a_ values: −COOH group of cephem moiety (p*K*_a_ = 1.9), −NH_2_ group of the aminothiazole moiety (p*K*_a_ = 3.3), and =N–OH group of the oxime moiety (p*K*_a_ = 9.9) (Figure 1B) [14]. According to the Henderson-Hasselbalch equation, the ‒COOH group of the cephem moiety (p*K*_a_ = 1.9) is almost completely ionized over about pH 4.0, likely causing the observed increased aqueous solubility of cefdinir over about pH 4.0. The physicochemical properties of cefdinir, including its p*K*_a_ values (Figure 1B), seem to contribute to the pH-dependent solubility, as shown in Figure 2. The CSD1, CSD2, and CSD3 formulations commonly gave at least 9-fold higher water solubility of cefdinir compared with that of cefdinir itself (Figure 3). Among the tested hydrophilic polymers, CMC-Na resulted in higher aqueous solubility of cefdinir in the CSD formulation or as a physical mixture.

The amorphous state of cefdinir and its molecular dispersion in the hydrophilic carrier may lead to the observed increased water solubility. The polymer-drug interactions may mainly be the result of hydrogen bond formation and/or hydrophobic interactions and the optimum polymer selection is a very critical point to enhance the solubility and dissolution of a poorly water-soluble drug [15]. Drugs can be molecularly dispersed and show a high wettability in the hydrophilic carrier [6].

In the DSC analysis, cefdinir showed a specific exothermal peak at 225.2 °C (Figure 4A). This was in accordance with the previously reported DSC curve of cefdinir anhydrous [15,16]. However, that exothermal peak observed in cefdinir was hardly observed in the CSDs (CSD1, CSD2, and CSD3), implying the amorphization of cefdinir in the CSDs. Furthermore, the sharp diffraction peaks of cefdinir crystalline form in the p-XRD analysis (Figure 5A) were absent in CSDs (Figure 5B–D), indicating its amorphization. Taking together the DSC and p-XRD analysis results, all the tested hydrophilic polymers can alter the state of cefdinir from a crystalline to an amorphous form efficiently with the current preparation method (at 1:1 weight ratio between drug and polymer).

In the SEM photography, the CSDs (CSD1–3) composed of hydrophilic polymers and cefdinir and prepared by the spray-drying method presented similar morphologies with rough surfaced spherical particles. In the particle size distribution, the order of overall particle sizes was CSD1 > CSD3 > CSD2, as shown in Figure 6B–D.

As exhibited in Figure 7, the CSDs significantly showed a higher percentage of drug release dissolution compared with that of cefdinir in all the tested media. Thus, the results clearly imply that higher dissolution occurred with the CSDs composed of hydrophilic polymer and the enhanced aqueous solubility can be attributed to this (Figure 3). Among CSD formulations, CSD3 exhibited the most immediate drug release profile. The lower and steady increase of dissolution from CSD1 and CSD2 may be due to the slow release of drug from the capsule though it was commonly proved to be highly soluble in water. To demonstrate the differences in dissolution profiles of CSDs, the viscosity of the aqueous dispersion of CSD was also measured. The viscosities of CSD1 and CSD2 were 2.8 and 2.7-fold higher than that of CSD3, respectively. This result indicates that the CSD1 and CSD2 filled in a gelatin capsule would be disintegrated slower than CSD3 with lower viscosity in the dissolution media. In other words, CSD1 and CSD2 which form stronger aggregates after exposing to aqueous environment took longer to disintegrate, and showed slow and steady dissolution curves, compared with CSD3 [17]. This difference in viscosity would be a result of the intrinsic properties of the hydrophilic polymers. HPMC and CMC-Na used in this study are water-swellable polymers, and they are more viscous than PVP K30, that is freely water-soluble polymer, in aqueous solution [18]. Compared CSD1 with CSD2, CSD1 resulted in lower dissolution rate compared with CSD2, due to its large particle size and high viscosity.

In particular, the in vivo oral absorption (presented as AUC_inf_) was significantly higher by 4.30-, 6.77- and 3.01-fold for CSD1, CSD2, and CSD3, respectively, compared with cefdinir suspension, in rats (Figure 9 and Table 2). The solubility data (Figure 3) and dissolution profiles (Figure 7) indicate that the higher solubility and dissolution characteristics in the developed CSDs formulations improved oral absorption of cefdinir in the gastrointestinal tract, compared with the cefdinir group. Comparing the solubility, dissolution and viscosity data of the CSDs in vitro, CSD2 showed the best solubility and high viscosity with a moderate dissolution rate among the three CSDs. As expected, the CSD2 formulation exhibited the most enhanced absorption in vivo among the three formulations.

Comparing the mucoadhesive properties of the polymers applied in this study, CMC-Na is known to have a higher degree of mucoadhesion than that of HPMC with the application for various formulations [19,20,21]. In contrast, PVP showed low or negligible mucoadhesive characteristics [22]. Taken together, it could be inferred that the high mucoadhesiveness and viscosity of CSD2 based on CMC-Na may lead to the longer retention of cefdinir in the upper gastrointestinal tract, and result in an improved oral bioavailability. In this investigation, other factors (i.e., drug permeation across the intestinal epithelium) may also contribute to determine oral bioavailability. Therefore, all of these in vitro results can be used to explain improved oral bioavailability of cefdinir by CSD formulations.

## 4. Materials and Methods 

### 4.1. Reagents and Materials

Cefdinir anhydrous (purity 97.5%, Figure 1) was purchased from Aurobindo Pharmaceutical Ltd. (Telangana, India). PVP K30, HPMC 60SH, and carboxymethylcellulose-Na 7LF (CMC-Na) were obtained from BASF Chemical Co., Ltd. (Ludwigshafen, Germany), Shin-Etsu Chemical Co., Ltd. (Tokyo, Japan), and Ashland Industries Europe GmbH (Schaffhausen, Switzerland), respectively. High performance liquid chromatography (HPLC) grade methanol was purchased from Fisher Scientific (Piscataway, NJ, USA). All other chemicals were of reagent grade and were used without further purification.

### 4.2. pH-Dependent Solubility of Cefdinir

Excess cefdinir (about 50 mg) were added to water (10 mL) and a series of aqueous media with pH values ranging from 1.2–8.0 (adjusted with 0.1 N HCl, diluted phosphoric acid, and/or NaOH solution). They were shaken in a water bath at 25 °C for 24 h and centrifuged at 3000× *g* for 10 min (LZ-1730R, LABOGENE, Seoul, Korea). The supernatant was diluted with methanol and filtered through a membrane filter (0.45 μm pore size). The resulting solution (10 μL) was analysed by the HPLC method mentioned below.

### 4.3. HPLC Analytical Condition of In Vitro Studies

Sample analysis was performed using a Shimadzu HPLC system consisting of LC-20AT pump, SIL-20AC autoinjector, CBM-20A communication bus module, CTO-20A column oven, and SPD-M20A diode array detector (Shimadzu, Kyoto, Japan). The separation of cefdinir was performed on a reverse phase C18 column (250 mm × 4.6 mm, 5 μm; Shiseido, Tokyo, Japan). The mobile phase consisted of 1 M tetramethylammonium hydroxide solution, water, 0.1 M ethylenediaminetetraacetic acid, and methanol (1.40:92.40:0.04:6.16, *v*/*v*/*v*/*v*) (pH 5, adjusted with diluted phosphoric acid), at a flow rate of 1.0 mL/min by modifying method in United States Pharmacopoeia (USP) (2010) [23]. The column and autosampler tray were maintained at 40 °C and 4 °C, respectively. The analytical run time was 10 min. UV detection was monitored at 254 nm and injection volume of sample was 10 μL. Data acquisition and processing were carried out using the Shimadzu LC Solution software.

### 4.4. Preparation of CSDs

CSDs were prepared by using a lab-scale spray dryer (Mini B-290, Buchi, Flawil, Switzerland). In our preliminary study, it was found that some physicochemical properties of CSDs composed of HPMC or CMC-Na with polymer/drug ratio of 3:1 were not adequate, and gave a lower yield value (data not shown). Therefore, the solid dispersion formulation compositions listed in Table 1 were applied. Cefdinir (1 g) was dissolved in methanol (100 mL) and the same amount of hydrophilic polymer was dissolved in water (100 mL). Then, the hydrophilic polymer solution was added to the cefdinir solution under magnetic stirring until a homogeneous solution was formed. This prepared solution was then directly spray dried with a nozzle of 0.7 mm diameter, and a flow rate of 2.5 mL/min provided by a peristaltic pump. The inlet and outlet temperatures were maintained at 120 °C and 60 °C, respectively. The spray pressure was 5 kg/cm^2^ and the aspiration setting in the vessel was at −23 mbar. In the preparation of physical mixtures, the cefdinir and each hydrophilic polymer were accurately weighed at the ratio of 1:1, and then mixed in a mortar to form a homogeneous mixture.

### 4.5. Characterization of the Prepared CSDs

The characteristics of the prepared CSDs were investigated by solid-state studies. The thermal properties were investigated using differential scanning calorimetry (DSC) with a DSC-Q10 instrument (TA Instruments, New Castle, DE, USA). About 4 mg of each sample was placed into a sealed aluminium pan before heating under a nitrogen flow (20 mL/min) at a heating rate of 10 °C/min from 25 °C to 250 °C (DSC).

The pXRD patterns were obtained by a powder X-ray diffraction system (D/Max-2500, Rigaku, Tokyo, Japan) with Ni-filtered Cu-Kα radiation at 30 mA and 40 kV. The data were acquired over a range of 2θ from 5° to 40°. The angular step size was 0.02° at a rate of 2°/min.

The morphology of CSDs, cefdinir, and physical mixtures were obtained by field emission SEM (FE-SEM; S-4300 SE, Hitachi Ltd., Tokyo, Japan). To test solubility of CSD in water, an excess amount of CSD was added to 5 mL of water in a glass bottle and stirred for 24 h at room temperature. Then, an aliquot (3 mL) of the solution was withdrawn and centrifuged at 3000× *g* for 10 min (LZ-1730R, LABOGENE). The supernatant was diluted with methanol and analyzed by the HPLC method mentioned above. In addition, viscosity of solid dispersion solutions was measured. For the preparation of 2% CSD solution, the 100 mg of each CSD was dissolved in 50 mL water and it was vigorously shaken for 15 min to obtain a transparent solution. The viscosity was measured using a digital viscometer (Model LVDV II+; Brookfield, Middleboro, MA, USA) equipped with a S34 spindle at a shear rate of 20 rpm and a temperature of 25 °C.

### 4.6. Dissolution Study

Considering that the solubility of cefdinir was markedly increased over pH 4.0 with pH-dependent solubility, the dissolution test was performed using USP (34) dissolution apparatus II with 0.1 N HCl solution (pH 1.2), acetate buffered solution (pH 4.0), and water as the dissolution media (900 mL) at 37 ± 0.5 °C. Speed of the paddle was adjusted to 50 rpm. CSDs or cefdinir powder filled in a gelatin capsule shell (#0), equivalent to cefdinir amount of 100 mg, was prepared and placed into a dissolution tester (Varian VK7000, Varian Inc., Santa Clara, CA, USA) [24,25]. At pre-determined intervals, aliquot (5 mL) of the media was collected and filtered through a membrane filter (0.45 μm pore size). The concentration of cefdinir in the filtrate was determined by the HPLC method.

### 4.7. In Vivo Oral Pharmacokinetic Study in Rats

The oral pharmacokinetics of developed three suspended formulations of CSD1, CSD2, and CSD3 in water was evaluated at a dose of 2 mg/kg of cefdinir in fasted male Sprague Dawley (SD) rats, and compared with that of cefdinir suspension in water (as a control group). All animal care and procedures were conducted following the Guiding Principles in the Use of Animals in Toxicology, as adopted in 1989, revised in 1999, and amended in 2008 by the Society of Toxicology (SOT, 2008). The protocols for the animal studies were also approved by the Institute of Laboratory Animal Resources of Inje University. Male SD rats weighing 280–300 g were fasted for 18 h prior to the experiments but were allowed free access to water. Under anesthetization with Zoletil (20 mg/kg, intramuscular injection), cannulation with polyethylene tubing (PE-50; Clay Adams, Parsippany, NJ, USA) to femoral artery and vein was performed as described in our previous study [26]. After oral administration of cefdinir in the cannulated rats, 150 μL blood was taken from the femoral artery at 0, 0.25, 0.5, 1, 1.5, 2, 3, 4, 6, and 8 h. The collected blood samples were centrifuged at 12,000× *g* at 4 °C for 10 min and the supernatant of plasma was obtained and stored at −80 °C.

### 4.8. Plasma Sample Preparation and Bioanalytical Conditions by LC-MS/MS

For the sample preparation, 50 μL plasma was mixed with 100 μL internal standard (IS) solution (in methanol) for deproteinization. After vortex-mixing, centrifugation at 12,000× *g* at 4 °C for 10 min was performed, and the clear supernatant was obtained for the cefdinir analysis. Cefdinir concentrations in plasma were determined by our previous bio-analytical method reported using a liquid chromatography tandem mass spectrometry (LC-MS/MS) [27]. Briefly, the LC-MS/MS system consisted of an Agilent HPLC and Agilent 6460 QQQ mass spectrometer with an ESI^+^ Agilent Jet Stream ion source (Agilent Technologies, Santa Clara, CA, USA). Synergi 4μ polar-RP 80A column (150 mm × 2.0 mm, 4 μm, Phenomenex, Torrance, CA, USA) was applied to separate cefdinir and cefadroxil (IS) from endogenous plasma substances. The mobile phase was the mixture of 0.1% formic acid and methanol (65:35, *v*/*v*) and the flow rate was 0.2 mL/min. In the detection system, multiple reaction monitoring (MRM) in the positive electrospray ionization (ESI^+^) mode were selected for cefdinir and IS, for which the parent ion to production ion transitions were as follows: cefdinir, 396.1→227.2; IS, 364.2→208.0. The data were acquired and processed using the Mass Hunter software (version A.02.00; Agilent Technologies).

### 4.9. Pharmacokinetic Data and Statistical Analysis

The plasma concentration levels for each formulation were analyzed using Winnonlin 5.0.1 (Pharsight, Cary, NC, USA) to generate the following pharmacokinetic parameters: the total area under the plasma concentration–time curve from time zero to time infinity (AUC_inf_); the terminal half-life (t_1/2_). The peak plasma concentration (C_max_) and time to reach C_max_ (T_max_) were read directly from the observed data. For comparison, the relative oral bioavailability (BA) was further calculated by dividing AUC after oral administration of CDSs by AUC after oral administration of cefdinir powder. To indicate statistically significant differences, *p*-value of less than 0.05 was considered based on a *t*-test between two means for unpaired data or a Duncan’s multiple range test posteriori analysis of variance (ANOVA) among three means for unpaired data. All data were expressed as mean ± standard deviation.

## 5. Conclusions

CSDs based on hydrophilic polymer were developed for the enhanced dissolution and in vivo oral absorption of the antibiotic drug cefdinir. The developed CSDs commonly resulted in about 9.0-fold higher solubility of cefdinir and a significantly improved dissolution profile in water at pH 1.2, compared with cefdinir crystalline form. The in vivo oral absorption (presented as AUC) was greatly increased by 4.30, 6.77, and 3.01-fold for CSD1, CSD2, and CSD3, respectively, compared with that of cefdinir suspension. The main reasons for the higher absorption of CSD2 are likely the controlled drug release and high mucoadhesiveness. Thus, CSD2 based on CMC-Na would be a promising formulation to enhance dissolution and bioavailability of cefdinir.

## Figures and Tables

**Table 1:** Composition of the CSDs developed in this study.

Ingredients (g or mL)	CSD1	CSD2	CSD3
Cefdinir anhydrous	1	1	1
HPMC	1	-	-
CMC-Na	-	1	-
PVP K30	-	-	1
Methanol	100	100	100
Water	100	100	100

HPMC, hydroxypropylmethylcellulose; CMC-Na, sodium carboxymethylcellulose; PVP, polyvinylpyrrolidone.

**Table 2:** Pharmacokinetic parameters of cefdinir after oral administration of cefdinir, CSD1, CSD2, and CSD3, at a dose of 2 mg/kg in rats (*n* = 3–4).

Pharmacokinetic Parameters	Cefdinir	CSD1	CSD2	CSD3
T_max_ (min)	240 ± 0	240 ± 0	210 ± 60	240 ± 0
C_max_ (μg/mL)	0.305 ± 0.011	0.769 ± 0.358 *	1.272 ± 0.538 *	0.525 ± 0.105 *
AUC_inf_ (μg∙min/mL)	152.4 ± 17.3	799.8 ± 345.6 *	1031.6 ± 683.1 *	459.1 ± 118.1 *
t_1/2_ (min)	236.6 ± 64.1	655.1 ± 172.7 *	489.4 ± 254.2	526.6 ± 189.9
Relative BA (%) ^a^	-	429.9	676.9	301.2

^a^ Bioavailability (BA) = AUC_inf,CSD formulation_/AUC_inf,powder_ × 100; * *p* < 0.05, compared with cefdinir group by Duncan’s multiple range test posteriori analysis of variance (ANOVA). T_max_ time to reach C_max_; C_max_, peak plasma concentration; AUC_inf_, the total area under the plasma concentration–time curve from time zero to time infinity; t_1/2_, terminal half-life.
